# Case Report: Unusual Presentations of Loss-of-Function Mutations of the Calcium-Sensing Receptor

**DOI:** 10.3389/fmed.2021.809067

**Published:** 2022-01-24

**Authors:** Serena Palmieri, Giorgia Grassi, Vito Guarnieri, Iacopo Chiodini, Maura Arosio, Cristina Eller-Vainicher

**Affiliations:** ^1^Unit of Endocrinology, Fondazione Istituto di Ricovero e Cura a Carattere Scientifico Ca' Granda—Ospedale Maggiore Policlinico, Milan, Italy; ^2^Division of Medical Genetics, Fondazione IRCCS Ospedale Casa Sollievo della Sofferenza, San Giovanni Rotondo (FG), Milan, Italy; ^3^Department of Endocrine and Metabolic Diseases, IRCCS, Istituto Auxologico Italiano, Milan, Italy; ^4^Departments of Medical Biotechnology and Translational Medicine, University of Milan, Milan, Italy; ^5^Department of Clinical Sciences and Community Health, University of Milan, Milan, Italy

**Keywords:** cinacalcet, hyperparathyroidism, CaSR, FHH, NSHPT

## Abstract

**Background:**

In recent years, heterozygous loss-of-function mutations of the Calcium Sensing Receptor gene (CaSR) were implicated in different hypercalcemic syndromes besides familial hypocalciuric hypercalcemia (FHH), including neonatal severe primary hyperparathyroidism (NSHPT) and primary hyperparathyroidism (PHPT).

**Cases presentation:**

Here we describe two unusual presentations of heterozygous inactivating CaSR mutations. Case 1: a case of NSHPT due to a *de novo*, p.(ArgR185Gln) CaSR mutation and successfully treated with cinacalcet monotherapy for 8 years until definitive surgical resolution. Case 2: a 37 years-old woman with PHPT complicated with hypercalcemia and nephrocalcinosis with a novel heterozygous p.(Pro393Arg) CaSR mutation and cured with parathyroidectomy.

**Conclusions:**

These cases reinforce the fact that the clinical spectrum of inactivating mutations of the CaSR has widened and, although carrying a mutation suggestive of FHH, some patients may have different clinical phenotypes and complications requiring individualized therapies.

## Introduction

The Calcium-Sensing Receptor (CaSR), a plasma membrane G protein-coupled receptor, provides the major mechanism for the detection of extracellular calcium concentration in several cell types and modulates parathyroid hormone (PTH) release in the parathyroid glands and urinary calcium reabsorption and excretion in the kidney (1–5). The central role of the CaSR in calcium homeostasis has been highlighted by the identification of CaSR gene mutations (chromosome 3q21.1) (5). Heterozygous loss-of-function mutations of the CaSR, or the downstream Gα11 and AP2σ signaling proteins, lead to hypercalcemic disorders and cause familial hypocalciuric hypercalcemia type 1 (FHH1, MIM #145980), type 2 (FHH2, MIM #145981) and type 3 (FHH3, MIM #600740), respectively (2, 3). Neonatal severe primary hyperparathyroidism (NSHPT, MIM #239200) tipically results from CaSR loss-of-function mutations inherited in an autosomal recessive manner from homozygosity or compound heterozygous state or from a *de novo* mutational event with an inherited mutant parental allele (2, 3). However, in some cases, heterozygous loss-of-function CaSR mutations have been described in patients with NSHPT (1, 6, 7). Besides gene dosage, other factors such as a dominant negative effect, paternal inheritance and *de novo* mutations have a role in the lack of a clear genotype/phenotype correlation (6). Moreover, rare associations of heterozygous loss-of-function CaSR mutations and primary hyperparathyroidism (PHPT) with both hyperplasia and/or adenoma of the parathyroid glands have been described and in some of these cases, patients clearly experienced clinical benefit and/or complete biochemical remission after parathyroid surgery (PTx) (7–13). Therefore, the concept that a heterozygous loss-of-function CaSR mutation in a patient with hypercalcemia is associated with a “benign” phenotype that does not benefit from medical or surgical management needs to be updated in order to offer the most appropriate therapeutic approach to each individual patient.

Here we describe two unusual presentations of heterozygous loss-of-function CaSR mutations: a case of NSHPT successfully treated with long term cinacalcet monotherapy until definitive surgical resolution and a case of complicated PHPT with heterozygous CaSR mutation cured with PTx.

## Case Description 1

**Figure 1:**
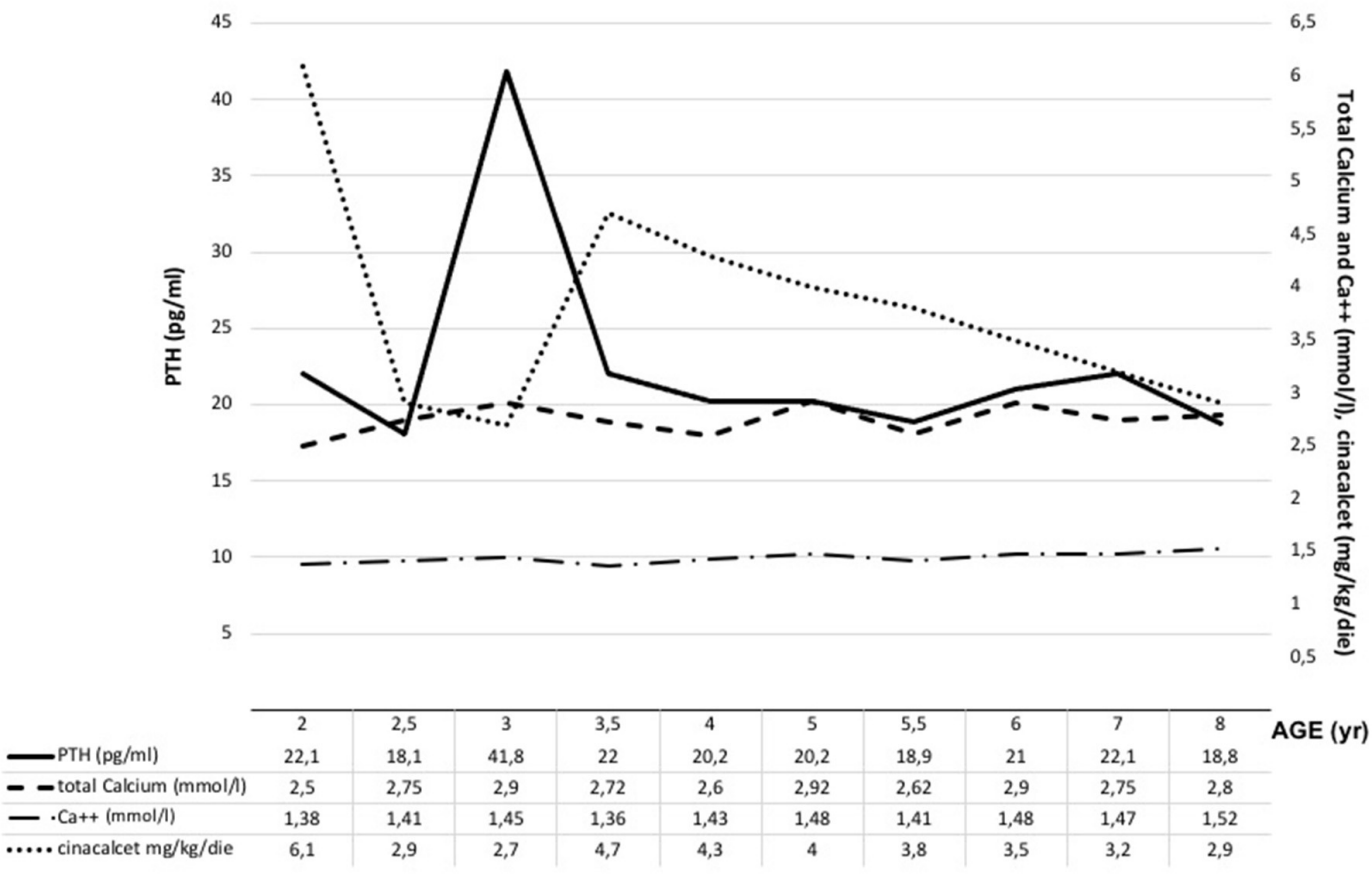
Biochemical data and cinacalcet doses. Ca^2+^, ionized calcium; PTH parathyroid hormone.

**Figure 2:**
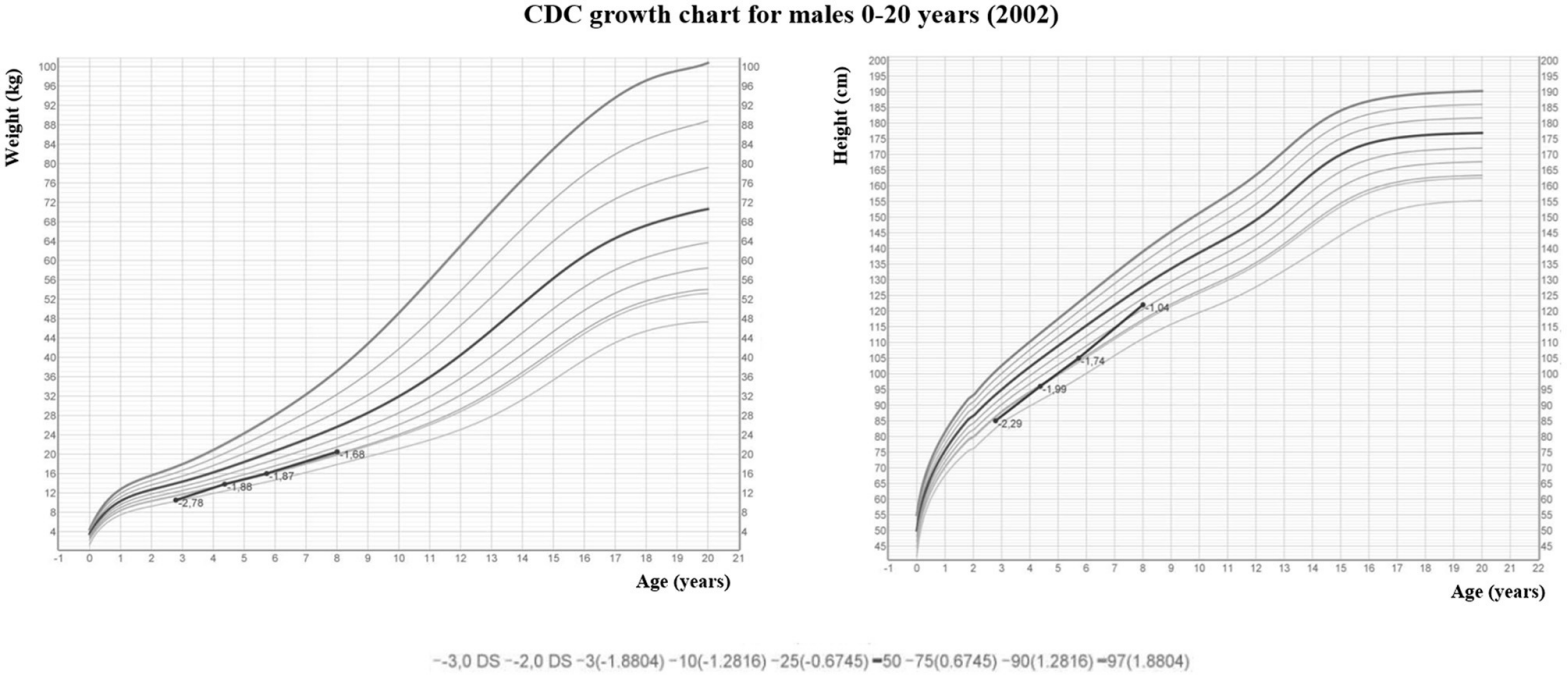
Growth curves of Case 1.

The male infant was born at term by spontaneous vaginal delivery after a normal pregnancy. The child appeared well initially, but, within hours, experienced hypotonia and severe respiratory distress. He was immediately transferred to the neonatal intensive care unit where he was intubated for surfactant administration with little benefit. Routine laboratory testing revealed markedly elevated serum levels of total calcium corrected to albumin levels (_adj_Ca, 3.75 mmol/L; normal range for age 2.10–2.55 mmol/L), ionized calcium (Ca^2+^, 1.96 mmol/L, normal range for age 1.13–1.32 mmol/L) and PTH (700 pg/mL; normal range for age 10.0–65.0 pg/mL). Other biochemical data were creatinine 0.16 mg/dl (normal range for age 0.31–0.88), phosphate 4.2 mg/dl (normal range for age 4.5–6.7 mg/dl), magnesium 2.5 mg/dl (normal range for age 1.8–2.4 mg/dl), alkaline phosphatase 363 U/l (normal range for age 100–400 U/l), 24-h urine calcium (24 hUCa) 34 mg/24 h. Both parents had calcium levels within the normal range and were unrelated, moreover the family history was unremarkable. NSHPT was confirmed by the finding of a previously reported heterozygous CaSR missense mutation at nucleotide position 554 (c.554G > A) in exon 4, leading to a substitution of arginine by glutamine at codon 185 [p.(Arg185Gln)] (14–17). This mutation was absent in both parents indicating a *de novo* mutation. For the management of the hypercalcemia, the patient initially received intravenous fluids and furosemide without significant improvement. Therefore, at 3 weeks of age (January 2011), the patient underwent exploratory neck surgery with the aim of performing a subtotal parathyroidectomy. Pre-surgery parathyroid ultrasound showed two hypoechoic nodules of 3 and 4 mm compatible with lower parathyroids but only one hypercellulated parathyroid gland (left superior) without cellular atypia was removed. For the persistence of symptomatic hypercalcemia, after obtaining informed consent from the parents, cinacalcet was started (initially 20 mg/m^2^/day divided in two oral doses). Cinacalcet treatment led to reductions in both serum concentrations of _adj_Ca and Ca^2+^ (3.1 and 1.55 mmol/L, respectively) with consequent clinical improvement. During hospitalization, cinacalcet was gradually increased up to 115 mg/m^2^/day divided into 2 doses until the calcium levels stabilized within the high-normal range. The only side effect that occurred was vomiting, progressively improved over time and definitively resolved with co-administration of thickened milk. The patient was discharged home at 5 months of life (May 2011, pre-discharge _adj_Ca 2.63 mmol/L, cinacalcet dose 6.5 mg/kg/day). During subsequent outpatient visits, therapy was gradually increased to maintain the daily dosage around 6–6.5 mg/kg/day with optimal biochemical control. The patient, previously evaluated elsewhere, came to our attention at the age of 2 (December 2012). At that time, his therapy was cinacalcet 60 mg/day divided into 2 doses (6.1 mg/kg/day) with an _adj_Ca of 2.5 mmol/l, Ca^2+^ 1.38 mmol/l (normal: 1.13–1.32 mmol/l), PTH 22.1 pg/ml (normal: 6.5–36.8 pg/ml) and 25-hydroxyvitamin D 27 ng/ml (25OHD, normal: 30–120 ng/ml) (Figure 1). The child presented normal motor, intellectual and relational abilities but parents complained about poor appetite and nausea in their son. The patient's weight and length-for-age were at the lower growth percentiles (Figure 2). Tests of thyroid function, growth hormone and celiac disease were unremarkable. An attempt to reduce the cinacalcet dose was performed with subsequent poor biochemical control and no improvement in gastrointestinal symptoms (Figure 1). A diet that provided the age-recommended daily amount of calcium was set and the patient was supplemented with cholecalciferol in order to normalize 25OHD levels. The patient was successfully and safely managed with cinacalcet through 8 years, with medical and biochemical controls every 6–12 months (Figure 1). During the course of this treatment, _adj_Ca and PTH levels were maintained close to the normal limits in the absence of specific symptoms of hypercalcemia (Figure 1), with progressive improvement in the auxologic parameters (Figure 2). However, from 6 years of age, the patient complained progressive increase in nausea and difficulties in taking tablets twice-daily. In consideration of these adverse symptoms and of the poor knowledge of the long-term effects of cinacalcet in children, parathyroid surgery was re-evaluated as definitive therapy. Finally, at 8 years of age (March 2019), a new exploratory surgery of the neck was performed. The pre-surgery neck ultrasound showed a 20 mm right lesion compatible with a parathyroid gland. The surgical operation was challenging for the presence of multiple adhesions related to previous excision. A subtotal PTx was performed with removal of one hypercellulated parathyroid gland (right superior, 9 mm) without cellular atypia. Two days after surgery the patient developed asymptomatic hypocalcemia treated with calcium, calcitriol and cholecalciferol supplements. Surgery was complicated by transient bilateral recurrent laryngeal nerves injury, with bilateral median vocal cord paresis that completely recovered in 6 weeks after speech therapy. At 2 months from surgery, nausea was no longer present, appetite improved and the patient gained 1.3 kg. At 2 years follow up (August 2021), hypoparathyroidism was permanent but therapeutic compliance was excellent with no hypocalcemic symptoms and no difficulties in taking calcium and calcitriol supplements.

## Case Description 2

A 37-year-old female patient presented in the emergency department with fever and abdominal pain. The examinations revealed an obstructive right pyelonephritis and multiple calcium deposits in the pyramids of both kidneys as well as kidney stones. Her personal and family history were unremarkable except for a chronic autoimmune euthyroid thyroiditis and a previous saphenectomy. After right ureteral stenting, laboratory results revealed the presence of PHPT with hypercalciuria: _adj_Ca 3 mmol/L, normal 2.10–2.55 mmol/L; Ca^2+^ 1.65 mmol/L, normal 1.13–1.32 mmol/L; PTH 451 pg/mL, normal 10.0–65.0 pg/mL; phosphorus (P) 2.3 mg/dl, normal 2.7–4.5 mg/dl; 25OHD 15.7 ng/ml normal: 30–120 ng/ml; 24 hUCa 451 mg/24 h (7.7 mg/kg/day). Pituitary function tests, serum gastrin, chromogranin A, calcitonin, creatinine and urinary normetanephrine and metanephrine were normal. In addition to renal complications, the bone densitometry revealed radius and femoral reduction in bone mineral density (BMD, lumbar spine 0.946 g/cm^2^, Z-score−0.8; femoral neck 0.725 g/cm^2^, Z-score−0.9), moreover the patient complained of lack of appetite. A diet that provided the age-recommended daily amount of calcium was set and the patient was supplemented with cholecalciferol in order to normalize 25OHD levels. The neck ultrasound and 99mTc-sesta MIBI tomoscintigraphy were both negative. She underwent a surgical 4-gland exploration (May 2020): a right upper retrocarotid parathyroid adenoma was found and excised. The macroscopic appearance of the other three parathyroid glands was normal. The pathological examination confirmed a 10 mm parathyroid adenoma. After surgery, the result of the genetic test was available and a novel heterozygous CaSR missense variant of unknown significance at nucleotide position 1178 (c.1178C > G) in exon 4, predicting a substitution of proline by arginine at codon 393 [p.(Pro393Arg)] was found. Screening for CDC73 and MEN1 mutations was negative. After PTx, the patient's hypercalcemia promptly normalized and at 1 year follow up (May 2021), a normalization of all the parameters of calcium-phosphoric metabolism was observed (_adj_Ca 2.22 mmol/L, normal 2.10–2.55 mmol/L; Ca^2+^ 1.23 mmol/L, normal 1.13–1.32 mmol/L; PTH 46 pg/mL, normal 10.0–65.0 pg/mL; P 3.4 mg/dl, normal 2.7–4.5 mg/dl; 24 hUCa 232.5 mg/24 h−3.9 mg/kg/day). Unfortunately, due to the COVID-19 travel restrictions, we could not test the adult family members of the index case.

## Genetic Analysis

Genomic DNA of probands was extracted from peripheral white blood cells by standard techniques. Molecular screening of the entire CaSR, CDC73 and MEN1 coding sequence (including exon-intron boundaries) was performed by PCR amplification and direct sequencing as previously reported (18, 19).

## Discussion

In this report, we described the unusual occurrence of heterozygous missense mutations in the CaSR, which is typical for FHH, in two patients with biochemical and histological findings consistent with NSHPT and PHPT respectively. The clinical interest of these cases lies in the fact that the case 1 describes the longest medical management of a NSHPT patient with cinacalcet monotherapy after initial surgical failure and the second case describes a rare association between PHPT and FHH with biochemical normalization after PTx.

Neonatal Severe Primary Hyperparathyroidism (NSHPT) is a rare condition characterized by marked symptomatic hypercalcemia, diffuse parathyroid hyperplasia, respiratory distress, muscular hypotonia, intestinal dysmotility, failure to thrive and skeletal demineralization that presents shortly after birth (1). If untreated, NSHPT can be a devastating neurodevelopmental disorder with a mortality rate >50% (1). In NSHPT, treatment options include total or subtotal parathyroidectomy, infusions of bisphosphonates, intravenous hyper-hydration and diuretics (2, 3, 8). In the last years, cinacalcet, a positive allosteric activator of the CaSR, have been used in the management of NSHPT to increase the receptor's sensitivity to extracellular calcium (2, 14–17, 20–24). The success of cinacalcet therapy in this clinical setting depends on the location of the mutation on the CaSR gene and the residual function of the mutated receptor (2, 14–17, 20–24). The p.(Arg185Gln) mutation is located in the extracellular domain of the CaSR and is associated with severe hypercalcemia and/or NSHPT even in a heterozygotic state, due to its dominant negative properties (25, 26). Indeed, the 185Gln mutated CaSR heterodimerizes with the wild-type receptor reducing its functional reserve (25). *In vitro* studies showed that the p.(Arg185Gln) mutation responded to the allosteric modulator NPS R-568 exhibiting both increased sensitivity to extracellular calcium and plasma membrane expression (26). To date 5 cases of NSHPT due to heterozygous p.(Arg185Gln) CaSR mutation and successfully treated with cinacalcet have been published, with a maximum follow-up of 32 months (14–17) (Table 1). Our case 1 patient underwent subtotal and ineffective parathyroidectomy at 3 weeks of life, hence, in consideration of the published data for the specific mutation, cinacalcet was offered as the therapeutic option. However, as with bisphosphonates, there is still no consensus on the optimal dosages and possible adverse effects from long-term treatment with cinacalcet therapy in pediatric patients (15, 20). Our report confirms that cinacalcet works well as a monotherapy in heterozygous p.(Arg185Gln) NSHPT patients, even in new-borns. Indeed, as previously described for this specific mutation (14–17), our patient responded to cinacalcet monotherapy with improvement in serum calcium and PTH levels and, primarily, in clinical conditions. The relevance of our report is that cinacalcet therapy remained effective in our patient from birth to 8 years, without interruptions. Another successful long follow up cinacalcet therapy (6 years, maximum dosage 90 mg/day) has been described in an older child (6 year-old) with homozygous c.206G > A CaSR mutation and previously treated with multiple surgery and bisphosphonates (from 2 to 6 years) (27). These findings are important because cinacalcet is not approved for use in children under the age of 18 and pediatric clinical trials of cinacalcet were suspended after the death of a 14-year-old patient (28). FDA advised health care professionals to pay specific attention to hypocalcaemia, which may be life-threatening, and to the most common gastrointestinal (GI) side effects of cinacalcet like nausea, vomiting and diarrhea (28). As showed in Figure 1, we have regularly monitored calcium levels in our patient with no hypocalcaemia. In fact, we were unable to completely normalize calcium levels despite the high doses of cinacalcet used that were far in excess of what normally used in adults with primary or secondary hyperparathyroidism. This was true particularly in the youngest ages as also emerges from the comparison with the other similar cases described in the literature and detailed in Table 1. Therefore, it is possible that the CaSR mutation itself requires high therapeutic dosages but we cannot exclude an unexpected effect of age on cinacalcet metabolism. The therapeutic compliance of our patient has always been excellent and we excluded the possibility of a malabsorption. Regarding the GI side effects related to cinacalcet therapy, our patient experienced nausea and poor appetite that completely resolved after surgery. No GI side effects were reported in previously described NSHPT cases due to heterozygous p.(Arg185Gln) CaSR mutation and treated with cinacalcet but this is probably related to the short duration of the follow-up and/or the young age of the patients treated (14–17) (Table 1). An unresolved issue is whether our patient's GI symptoms were actually related to cinacalcet therapy or to hypercalcaemia itself, both removed after surgery. After 2 years from second surgery, hypoparathyroidism was permanent with excellent therapeutic compliance and no hypocalcemic symptoms. However, long-term outcome data from parathyroidectomized NSHPT remain to be evaluated.

**Table 1:** Literature review of NSHPT due to heterozygous R185Q CaSR mutation successfully treated with cinacalcet therapy.

**Reference**	**Initial therapies (before cinacalcet)**	**Start cinacalcet (age)**	Follow-up (time)^*^	**Cinacalcet dose** **(S/F/M)^**#**^**	**Cinacalcet side-effects**
Reh et al. (14)	Intravenous fluids, furosemide, pamidronate 0.5 mg/kg (single dose), cholecalciferol	3 weeks	16 months	S: 1.8 mg/kg/day (20 mg/m^2^/day) F/M: 30 mg/day	None
Gannon et al. (15)	Intravenous fluids, phosphate supplements, cholecalciferol	Within 3 weeks	17 months	S: 0.4 mg/kg/day (6 mg/m^2^/day) F/M: 9.6 mg/kg/day (202 mg/m^2^/day)	None
Fisher et al. (16)	Intravenous fluids, low Ca formula, pamidronate 0.5 mg/kg (single dose)	13 months	32 months	S: 3.7 mg/kg/day F: 2.4 mg/kg/day M: 7.4 mg/kg/day	None
Fisher et al. (16)	Intravenous fluids, phosphate supplements, low Ca formula	4 months	13 months	S: 2.0 mg/kg/day F/M: 2.7 mg/kg/day	None
Forman et al. (17)	Intravenous fluids, phosphate supplements	7 days	14 months	S: 0.4 mg/kg/day F/M: 5 mg/kg/day (112 mg/m^2^/day)	None
Palmieri S., 2021 (this work)	Intravenous fluids, furosemide, sub-total parathyroidectomy	1 month	98 months	S: 20 mg/m^2^/day F: 2.9 mg/kg/day M: 6.1 mg/kg/day	Vomiting (only at the start of the therapy), nausea and poor appetite

* *Cinacalcet therapy duration*

# *S, starting dose; F, final dose; M, maximum dose*.

The occurrence of both CaSR mutation and PHPT is infrequent with a 0.6–2.9% prevalence in series of consecutive PHPT patients (18, 29). Inactivating CaSR mutations have been identified both in isolated cases and hereditary cases of familial isolated HPT (FIHP) (9–11). These patients show different clinical and biochemical presentations (symptomatic hypercalcemia with variable degree of urinary calcium excretion), may develop typical PHPT complications and parathyroid tumors (ranging from multiglandular hyperplasia to single adenomas) with a variable response to surgical therapy (from reduced symptoms and calcium levels to complete biochemical normalization) (11–13, 30, 31). Given this variability, the role of CaSR mutations in the pathogenesis of acquired PHPT is unclear; however, no mutations were found in 200 healthy controls supporting the link between the two conditions apart few cases (18). Some authors hypothesized that germline CaSR mutations may promote parathyroid cell proliferation and susceptibility to additional potential specific genetic hits with development of monoclonal parathyroid lesions (32).

The p.(Pro393Arg) mutation which we described in Case 2 has not, to the best of our knowledge, been previously reported. Following the guidelines for interpretation of pathogenicity (33), and https://varsome.com/), this variant can be classified as “likely pathogenic”. The P393 residue lies into the “Venus-Flytrap” domain of the receptor, pretty adjacent to the Cysteine 395 which can be predicted to bond the Cys358 (32). Modifications of these Cysteines, as well as of the other ones (C60-C101 and C437-C449) impair the correct folding of the VFT domain and, thus, the overall ability of the receptor to bind the Ca^2+^ (32 and references herein). Thus, although in absence of any functional data, we could infer that the planar structure of the Arginine, at the variance with the classic “hinge” shape of the Proline, might strongly interfere the binding of the two Cys395-Cys358, thus making the receptor less sensitive to the Ca^2+^. Of particular interest in this patient is the presence of a functioning parathyroid adenoma and the complete biochemical normalization after PTx which are both rare event in CaSR loss of function mutations with sporadic cases reported in single patients or in FHIP kindreds (9, 14, 29, 34, 35). These findings are atypical for FHH, since PTx typically does not lead to resolution of hypercalcemia and FHH is not typically associated with adenoma formation (36) and despite also having a CaSR mutation, these patients benefited from PTx.

## Conclusions

Our knowledge of hypercalcemic disorders related to inactivating mutations of the CaSR has expanded. These cases show how, even in the presence of mutations typical for FHH, these patients required individualized therapies. In particular, we report the longest follow-up of cinacalcet monotherapy in a patient with NSHPT that was overall well tolerated (except for mild GI symptoms) without signs for loss of effectiveness over time, reinforcing the knowledge of this therapeutic option in this clinical context where the medical management is often challenging and surgery unsuccessful at a very young age. Moreover we described a novel CaSR mutation associated with hypercalciuria, renal calculi and a parathyroid adenoma with biochemical resolution after PTx, and, although more studies are required to confirm the pathogenetic role of this CaSR mutation in the pathogenesis of PHPT, it is important to recognize these singular cases given the potential benefit from surgical treatment in this context.

Unfortunately, case report studies have an intrinsic limitation given by the poor reproducibility of the clinical approach due to the uniqueness of the cases described and the mutations reported in the individual patient. More cases and, if possible systematic analyses, are needed to draw strong conclusions and recommend suitable treatment strategies for each individual case.

## Data Availability Statement

The raw data supporting the conclusions of this article will be made available by the authors, without undue reservation.

## Ethics Statement

Written informed consent to participate in this study was provided by the participants' legal guardian/next of kin. Written informed consent was obtained from the individual(s), and minor(s)' legal guardian/next of kin, for the publication of any potentially identifiable images or data included in this article.

## Author Contributions

SP was responsible for data collection and wrote the paper. GG collected data. VG conducted the genetic analysis. IC supervised and collected data. MA and CE-V supervised and wrote the paper. All authors contributed to the article and approved the submitted version.

## Funding

This research was partially funded by the Italian Ministry of Health Ricerca Corrente 2018–2021.

## Conflict of Interest

The authors declare that the research was conducted in the absence of any commercial or financial relationships that could be construed as a potential conflict of interest.

## Publisher's Note

All claims expressed in this article are solely those of the authors and do not necessarily represent those of their affiliated organizations, or those of the publisher, the editors and the reviewers. Any product that may be evaluated in this article, or claim that may be made by its manufacturer, is not guaranteed or endorsed by the publisher.
